# Phylogenetic Origins of Brain Organisers

**DOI:** 10.6064/2012/475017

**Published:** 2012-07-03

**Authors:** Ellen Robertshaw, Clemens Kiecker

**Affiliations:** MRC Centre for Developmental Neurobiology, King's College London, 4th Floor, New Hunt's House, Guy's Hospital Campus, London SE1 1UL, UK

## Abstract

The regionalisation of the nervous system begins early in embryogenesis, concomitant with the establishment of the anteroposterior (AP) and dorsoventral (DV) body axes. The molecular mechanisms that drive axis induction appear to be conserved throughout the animal kingdom and may be phylogenetically older than the emergence of bilateral symmetry. As a result of this process, groups of patterning genes that are equally well conserved are expressed at specific AP and DV coordinates of the embryo. In the emerging nervous system of vertebrate embryos, this initial pattern is refined by local signalling centres, *secondary organisers*, that regulate patterning, proliferation, and axonal pathfinding in adjacent neuroepithelium. The main secondary organisers for the AP neuraxis are the midbrain-hindbrain boundary, zona limitans intrathalamica, and anterior neural ridge and for the DV neuraxis the notochord, floor plate, and roof plate. A search for homologous secondary organisers in nonvertebrate lineages has led to controversy over their phylogenetic origins. Based on a recent study in hemichordates, it has been suggested that the AP secondary organisers evolved at the base of the deuterostome superphylum, earlier than previously thought. According to this view, the lack of signalling centres in some deuterostome lineages is likely to reflect a secondary loss due to adaptive processes. We propose that the relative evolutionary flexibility of secondary organisers has contributed to a broader morphological complexity of nervous systems in different clades.

## 1. Introduction

One of the major challenges in biology is to understand how the remarkable complexity of the vertebrate brain arises—both ontogenetically, as the individual organism develops, and phylogenetically, in the course of evolution. Over the last 30 years, developmental biologists have identified groups of orthologous genes that are expressed in comparable anteroposterior (AP) and dorsoventral (DV) arrangements in both protostome and deuterostome embryos, leading to the view that positional information is broadly conserved across the animal kingdom [1–3]. With respect to the AP axis of the nervous system, conserved patterns of gene expression have led to the idea that a “tripartite brain”—roughly corresponding to the forebrain, midbrain/hindbrain, and spinal cord of vertebrates—is a common feature of bilaterians [4, 5].

Meanwhile, vertebrate central nervous system (CNS) development was found to be regulated by local signalling centres, *secondary organisers*, that control patterning, proliferation, differentiation, and axon guidance in adjacent neuroectoderm by secreting intercellular signalling factors [6–9]. No clear evidence for equivalent secondary organisers has been found in protostomes yet. The search for their phylogenetic origin in nonvertebrate species has revealed possible conservation of some, but not necessarily all, of these organisers in some of the closer relatives of vertebrates. A recent study has identified three signalling centres in the embryo of the hemichordate *Saccoglossus kowalevskii* that are reminiscent of the three main secondary AP organisers in vertebrates, prompting a reevaluation of the origins of these patterning centres [10].

Here, we will review the evidence for the occurrence of secondary organisers in different nonvertebrate clades and briefly discuss two recent studies on organiser variability in closely related vertebrates. Based on these studies, we propose that the relative evolutionary flexibility of secondary organisers has allowed for the rapid adaptation and diversification of brain morphology.

## 2. Early Axial Patterning and the Primary Organiser

In vertebrates, a crude pattern is already imparted upon the nascent neural plate before and during gastrulation. DV identity is regulated by dose-dependent Bone morphogenetic protein (Bmp) signalling [11, 12] whereas posterior identity is induced by signalling factors of the Fibroblast growth factor (Fgf), retinoic acid, and Wnt families [13–17]. Wnts posteriorise the neural plate (and presumably also the mesoderm and endoderm [18]) dose-dependently in a manner that is consistent with classical models for AP axis formation proposed in the 1950s [19, 20].

*Bmp* and *Wnt* genes are present in all animals examined including nonbilaterians of the phyla Cnidaria and Ctenophora [21–28], and *Wnts* are even found in Porifera [29–33]. The Wnt/*β*-catenin signalling pathway regulates axial patterning in a large number of phyla including the Cnidaria, leading to the conclusion that its role in axis formation may reach back to the eumetazoan ancestor and that the Wnt-induced axis is the primary axis of animals [18, 34–38]. Somewhat at odds with this theory, no evidence for a global role of Wnts in axis formation has been identified in the well-characterised embryo of the fruit fly *Drosophila melanogaster*, although Wnt signalling regulates AP polarity locally within its parasegments. However, Wnt activity is required for posterior growth in the orthopteran cricket *Gryllus bimaculatus* [39], in the short-germ beetle *Tribolium castaneum* [40] and in the spider *Achaearanea tepidariorum* [41], suggesting that the axis-forming activity of Wnt signalling may have been lost secondarily in the highly derived long-germ dipteran *Drosophila*. Consistent with this, entire subfamilies of *Wnt* genes seem to have disappeared from some protostome lineages [42].

Dose-dependent Bmp signalling patterns the secondary (DV) axis in all bilaterians examined. However, whereas highest levels of Bmp signalling specify the ventral side of the chordate embryo, they specify the most dorsal cell fates in arthropods [18, 26, 43, 44], providing molecular support for E. Geoffroy Saint-Hilaire's almost 200 year-old proposal that the DV axis has been inverted in animal evolution [45–47]. Similar to the role of Wnts in AP axis formation, the role of Bmps in DV patterning is not restricted to the ectoderm: for example, an increasing gradient of Bmp signalling also patterns the mesoderm into notochord, somitic mesoderm, lateral plate mesoderm and blood progenitors [48]. Notably, asymmetric expression of *Bmps* and their inhibitors has also been found in cnidarians, raising the possibility that the origins of the secondary, Bmp-patterned axis also reaches back past the bilaterian ancestor [23, 49–52].

Based on these studies, it has been proposed that orthogonal gradients of Bmp and Wnt signalling activity are ancient features of axis specification that were already in place when the Bilateria emerged [18, 35]. Since their activities are not limited to neuroepithelial cells or even to the ectoderm, it is likely that these ancestral mechanisms were adopted later to convey AP and DV polarity to the early embryonic CNS in vertebrates. Remarkably, although a global Wnt gradient seems to be absent in *Drosophila*, the fly larva's imaginal discs—epithelial outpocketings that will form the appendages of the adult fly—are also patterned by orthogonal gradients of Bmp and Wnt signalling, suggesting that these gradients constitute a module that is used repeatedly to set up a Cartesian network of positional information [53].

In vertebrates, axis formation during gastrulation depends on *Spemann's organiser* (also called the *primary *or *gastrula organiser *or* the node *in amniotes). Transplantation studies in (and between) different vertebrate species demonstrated that this organiser has the capacity to induce an embryonic axis that is patterned along its AP and DV axes. Spemann's organiser expresses several Bmp/Transforming growth factor *β* (Tgf*β*) and Wnt inhibitors and these factors mimic the axis-inducing activities of the organiser in gain-of-function experiments. Thus, it appears that the major function of this primary organiser is to antagonise the Bmp and Wnt signals that define the primary and secondary axes of the organism [13, 54].

Spemann's organiser is strictly required for DV patterning [13, 55, 56], but its relevance for AP patterning is somewhat controversial. Microsurgical ablation of the organiser, or of organiser-derived tissue, in fish, chick, frog or mouse results in anterior truncations [57–60]. Similarly, anterior defects are observed in frog and fish embryos deficient for the early organiser factors *siamois* and *bozozok*, respectively [61–63]. However, extensive AP patterning has been observed in the absence of the organiser under different experimental conditions [40, 64–70]. There are different, not necessarily exclusive, explanations for the relative independence of AP patterning from organiser-derived signals.

1. *The organiser may express not only anteriorising growth factor inhibitors, but also posteriorising factors*. Indeed, expression of *Wnt3/3A* is found in the chordamesoderm, the posterior derivative of Spemann's organiser, in mouse and frog embryos [71, 72]. Thus, organiser ablation may remove both the anteriorising Wnt inhibitors and the posteriorising Wnts.
2. *Other signalling centres at the anterior end of the embryo may compensate for the loss of Spemann's organiser in AP patterning*. The anterior hypoblast/anterior visceral endoderm is an extraembryonic tissue that underlies the prospective forebrain region of amniote embryos before the onset of gastrulation. It expresses Wnt inhibitors and is required for anterior neural induction, consistent with a role as a non-Spemann anterior organiser [58, 73–77]. Furthermore, a signalling centre that expresses Wnt (and, at least in frog, also Bmp) inhibitors is found in the anterior ectoderm of frog and fish embryos [78–81].
3. *Posteriorising factors such as Wnts may simply be able to diffuse and establish a concentration gradient by themselves*, without a strict need for a countergradient of inhibitors [35, 70].

Notably, Wnt inhibitors are also found in cnidarians where they are expressed in a manner complementary to Wnts, indicating that Wnt inhibition was already a component of the molecular system that patterns the primary body axis before the emergence of bilateral symmetry [34, 82]. Localised expression of Bmp and Wnt inhibitors and of other factors characteristic of Spemann's organiser has recently been described in the cephalochordate amphioxus, a basal chordate, suggesting that Spemann-type AP- and DV-patterning centres could be ancestral chordate features [43]. The evolutionary origins of Spemann's organiser may be even older: Hans Meinhardt has proposed that the circular region that patterns the primary body axis in radially symmetric animals may have evolved into a Spemann-type organiser through the addition of a symmetry breaking event, resulting in an organising centre that elongates on one side of the bilaterian embryo [35].

In vertebrates, the initial crude pattern induced by Bmp and Wnt signalling during gastrulation is subsequently further refined through the formation of various secondary organisers at specific AP and DV positions.

## 3. Anteroposterior Patterning

### 3.1. Midbrain-Hindbrain Boundary

**Figure 1:**
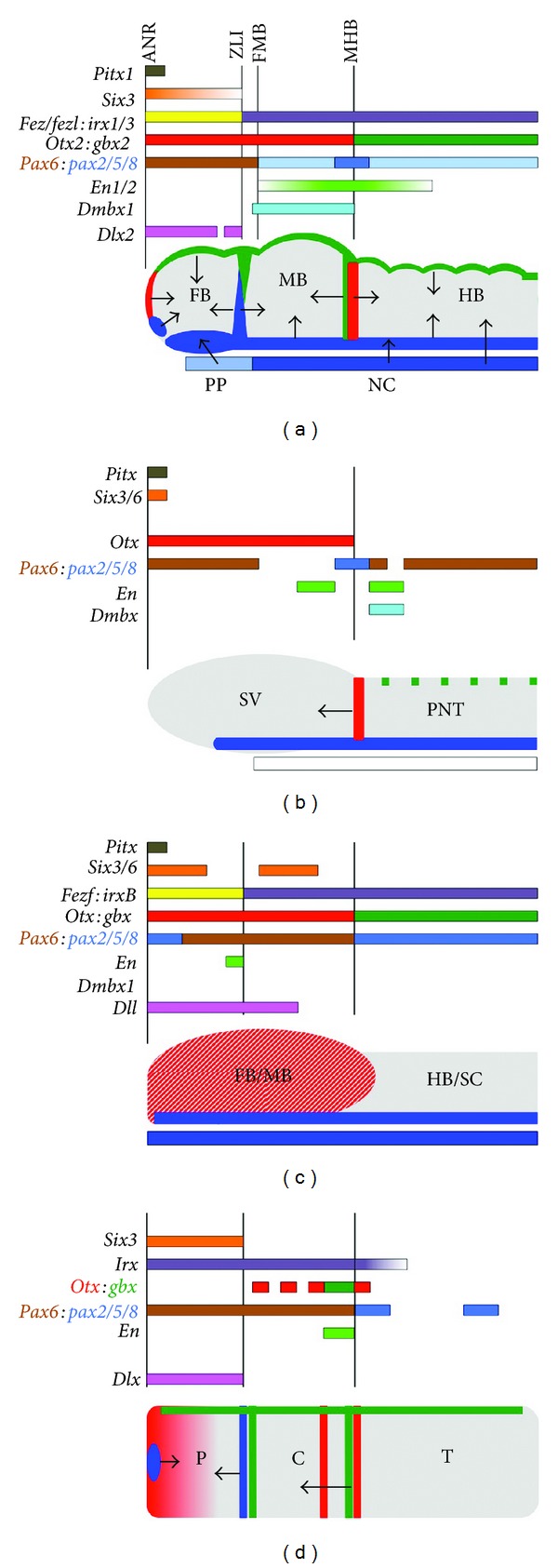
Expression of patterning genes and topography of secondary organisers in four deuterostome lineages. (a) Vertebrate neural tube, (b) ascidian nerve cord, (c) amphioxus nerve cord, and (d) *Saccoglossus kowalevskii* larva. ANR: anterior neural ridge, C: collar, FB: forebrain, FMB: forebrain-midbrain boundary, HB: hindbrain, MB: midbrain, MHB: midbrain-hindbrain boundary, NC: notochord, P: proboscis, PNT: posterior neural tube, PP: prechordal plate, SC: spinal cord, SV: sensory vesicle, T: trunk, and ZLI: zona limitans intrathalamica. Signalling centres express *Bmps* and *Wnts* (green), *Fgfs* (red) or *Hh* (blue); arrows indicate that a signalling function has been experimentally demonstrated. For gene names and references see text.

Perhaps the best studied of the vertebrate secondary organisers is the midbrain-hindbrain boundary (MHB), also known as *the isthmus*, that is crucial for the patterning of the midbrain and anterior hindbrain. Two signalling factors, Wnt1 and *Fgf8*, are expressed at the MHB, and various lines of evidence indicate that *Fgf8* is the main factor that mediates its signalling function [7, 83–85]. The MHB is positioned directly by the Wnt signal that posteriorises the neural plate [86], by the expression of *Pax2/5/8* and *Lmx1b* (encoding transcription factors) in the future MHB region, and then by the mutual repression between the homeobox genes *Otx2* in the prospective midbrain and *Gbx2* in the prospective hindbrain. Downstream of the organiser signal, homeobox genes of the *Engrailed* (*En*) family and negative Fgf feedback inhibitors of the Sprouty family are induced in expression gradients that are likely to reflect the graded distribution of Fgf ligands (Figure 1(a)).

**Figure 2:**
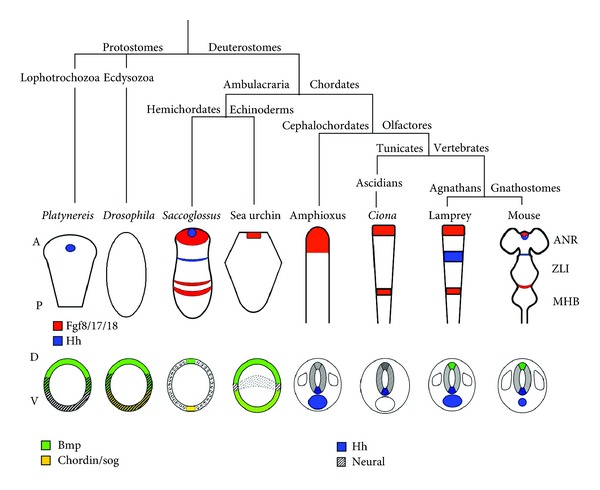
Phylogenetic tree leading to model organisms discussed in this paper. Signalling factor expression domains along the anteroposterior (AP) and dorsoventral (DV) axes are colour-coded.

Is a comparable signalling centre present in non-vertebrate phyla? Tunicates, also known as urochordates, are marine filter-feeders that are commonly regarded as the sister group of vertebrates (Figure 2). Their adult body plan is often very different from vertebrates since most tunicates are sac-like, sessile animals that live attached to rocks or the ocean floor. However, tunicate larvae swim freely and have a long tail with a notochord and a dorsal neural tube that enlarges anteriorly, forming a cerebral ganglion. Thus, tunicates are more similar to vertebrates at larval stages [87]. AP patterning genes are expressed in the tunicate CNS in an arrangement that is comparable to vertebrates (Figure 1(b)) [88–90]. In the tunicate *Ciona intestinalis*, orthologues of the vertebrate MHB genes *Fgf8* and *Pax2/5/8 *are expressed between the posterior sensory vesicle (PSV) and the visceral ganglion (VG) in the “neck region” of the larva [91]. The knockdown of *Ciona Fgf8/17/18* using morpholino antisense oligonucleotides resulted in gene expression changes in this region that are indicative of a loss of the “neck” and a posterior expansion of the PSV, demonstrating that the PSV/VG interface patterns this region by secreting Fgfs [92]. These findings suggest that an Fgf-dependent secondary organiser similar to the vertebrate MHB is present in the *Ciona* neck region.

Cephalochordates represent a sister group of the Olfactores (vertebrates and tunicates) that are also characterised by a notochord and a dorsal nerve cord. In amphioxus, *otx* and *gbx* are expressed in a pattern highly reminiscent of their vertebrate counterparts; however, *pax2/5/8* and the factors that mediate the organiser function of the vertebrate MHB, Fgf8 and Wnt1, are not specifically expressed at the *otx/gbx* interface [93–97]. Furthermore, no expression of the orthologue of the vertebrate midbrain marker *Dmbx1* is detected and expression of *en* is induced comparably late in the amphioxus nerve cord (Figure 1(c)) [91, 98]. Thus, although an *otx/gbx* interface is present, there is no evidence for an MHB-like organiser in amphixous, presumably because it has been lost secondarily in the amphioxus lineage (Figure 2).

The structure of the nervous system of the hemichordate acorn worm *Saccoglossus kowalevskii* differs considerably from that of vertebrates: instead of a centralised CNS like the chordate dorsal nerve cord, *Saccoglossus* has a diffuse network of neurons distributed throughout the ectoderm [99, 100]. Nonetheless, AP-patterning genes are expressed in three domains—proboscis, collar and trunk—in line with the idea of a tripartite body plan [101]. The authors of a recent study found expression of both *wnt1* and *fgf8/17/18* at the boundary between the collar and trunk of the *Saccoglossus* embryo, at an interface between *otx* and *gbx* expression. This area is also positive for the expression of *pax2/5/8* and the Fgf target gene *en* (Figure 1(d)). Even the catecholaminergic differentiation marker tyrosine hydroxylase is found in the *en*-positive domain, a marker of dopaminergic midbrain neurons in vertebrates. Thus, gene expression and neuronal differentiation at the hemichordate collar/trunk boundary resembles that of the vertebrate MHB. Importantly, they also show that *en* becomes downregulated upon inhibition of *fgf8/17/18* signalling, implying that this signalling centre functions as an organiser of local patterning [10]. However, some differences between the trunk/collar boundary and the vertebrate MHB should be noted: an additional ring of *fgf8/17/18* expression is present anterior to the *wnt1 *domain and the *gbx* domain is flanked by *otx* expression on either side (Figure 1(d)). It is tempting to speculate that these two observations could be linked: as the *Otx2/Gbx2* interface determines the site of *Fgf8* expression in vertebrates [83–85], two *otx/gbx* interfaces in *Saccoglossus* could give rise to two rings of *fgf* expression. The presence of an MHB-like signalling centre in hemichordates suggests that this secondary organiser may not be an Olfactores innovation (Figure 2).

Some additional support for the notion of an MHB-like signalling centre in lower deuterostomes comes from the finding that a module of *fgf-pax2/5/8-sprouty* expression is also active in the lateral ectoderm of sea urchin embryos [102]. Sea urchins are echinoderms, a sister group of the hemichordates with a highly derived anatomy (Figure 2). A potential function of *Fgf* signalling in sea urchin neural patterning remains to be investigated.

Thus, an MHB-like secondary organiser may have been a feature of the deuterostome ancestor that has subsequently degenerated or been lost in some clades. But what about protostomes? The identification of a “tripartite brain” in *Drosophila* suggests that the deuterocerebral/tritocerebral interface is the equivalent of the vertebrate MHB region with an anterior domain that expresses the *otx* orthologue *orthodenticle* and a posterior domain that expresses the *gbx* orthologue *unplugged* [4]. A similar pattern has been found in the annelid *Platynereis dumilii* [5]. However, although *wingless* (*Drosophila wnt*) and *en* are expressed in this region, there is no evidence for the expression of a *Pax2/5/8* orthologue or of *Fgf*-related genes there [103]. Hence, no evidence for an MHB-like signalling centre has been found outside of the deuterostome superphylum (Figure 2). However, we cannot exclude at this point that this organiser was lost secondarily in the highly derived *Drosophila* embryo, similar to the proposed loss of the MHB in amphioxus.

### 3.2. Zona Limitans Intrathalamica

The interface between the thalamus and prethalamus, the *zona limitans intrathalamica* (ZLI), in the posterior vertebrate forebrain functions as an organiser that regulates the patterning and differentiation of this brain region by secreting the signalling factor Sonic hedgehog (Shh) [8, 9, 104, 105]. Similar to the positioning of the MHB by the mutual antagonism between *Otx2* and *Gbx2*, the ZLI appears to form at the interface between *Fez/Fez-like* expression anteriorly and *Irx* expression posteriorly (Figure 1(a)). It forms in a transverse domain that is negative for the expression of *Lunatic fringe*, which is expressed throughout the remainder of the forebrain, and also expresses the signalling factor *Wnt8b*. Shh from the ZLI induces the homeobox gene *Dlx2* in the prethalamus anteriorly, and *Sox14* and *Gbx2* in the rostral and caudal domains of the thalamus posteriorly, as a function of higher and lower doses of Shh, respectively. A ZLI is also found in lampreys, jawless vertebrates that lack *Shh* expression in other areas of the forebrain (see below, Figure 2) [106, 107].

Both *Ciona* and amphioxus seem to lack a ZLI-like expression domain of *hedgehog* (*hh*) in the anterior nerve cord (Figures 1(b) and 1(c)) [108, 109]. However, an expression domain of *hh* is found at the proboscis/collar boundary of *Saccoglossus*. Notably, this stripe of *hh* expression is also found in a *fringe*-negative, *otx/wnt8*-positive domain and at the interface between *fez* and *irx *expression, highly reminiscent of the vertebrate ZLI (Figure 1(d)) [10]. In zebrafish, ZLI formation depends on *otx* function [110], and Pani et al. demonstrate that the same holds true for the ZLI-like signalling centre in *Saccoglossus*. Finally, they also prove that this signalling centre functions in patterning: injection of morpholino antisense oligonucleotides against Hh results in a loss of the anterior marker *dlx*, similar to vertebrates [111].

Despite the lack of *hh* expression in the anterior nerve cord of amphioxus, the transcriptional network underlying ZLI positioning in vertebrates appears to be conserved in cephalochordates, as an anterior expression domain of *fez* and a posterior domain of *irx* can be found in amphioxus embryos (Figure 1(c)) [112]. To date, there has been no confirmation of a *hh*-secreting signalling centre within the anterior brain of *Drosophila* larvae. However, as in amphioxus, there is evidence to suggest that their forebrains retain a similar overall prepattern, including abutting domains of *fez* and *mirror* (a *Drosophila* homologue of *Irx*) [112].

### 3.3. Anterior Neural Ridge

The anterior neural ridge (ANR) at the rostral end of the vertebrate neural plate functions as a secondary organiser that induces the anterior forebrain via secretion of Wnt inhibitors and Fgf8 [6]. As the neural plate rolls up and closes to form the neural tube, this signalling centre morphs into a patch of cells at the anterior-most tip of the embryonic CNS that continues to express *Fgf8* (Figure 1(a)). These cells give rise to the commissural plate, a scaffold for the formation of the forebrain commissures. Correct specification of the telencephalon depends on *Fgf* signalling from this secondary organiser [113–115]. At later stages, a gradient of Fgf8 signalling from the commissural plate contributes to the arealisation of the neocortical primordium [116]. Although the ANR appears to be common to all vertebrates studied thus far, the molecular mechanisms mediating its patterning function may vary slightly; for example, evidence that Wnt inhibitors of the Secreted frizzled related protein family (Sfrps) are expressed within the mouse ANR is lacking to date. It is possible that the relative importance of different sources of Wnt inhibitors has shifted between different vertebrates: in zebrafish the main source of forebrain-inducing signals appears to be the ANR, in amphibians the prechordal plate is essential, and in amniotes both the anterior visceral endoderm and the prechordal plate are required for proper anterior patterning [6, 117].

Whereas Wnt inhibitors that are expressed at the anterior tip of the CNS have not been identified in amphioxus, *sfrp1/5* is expressed there in *Ciona *[118]. Conversely, no *fgf* expression is found in this region in *Ciona*, but *fgf8* is expressed widely throughout the anterior ectoderm of amphioxus (Figures 1(b) and 1(c)) [95–97]. A recent study has demonstrated a requirement for Fgf signalling in positioning the anterior border of the *Ciona* nerve cord; however, no localised ectodermal expression of *fgf* has been associated with this function [119]. Nevertheless, *pitx*,an orthologue of mouse* Ptx1 *which is expressed in structures originating from the ANR, marks the anterior boundary of *Ciona's* CNS, suggesting some genetic conservation in this region (Figure 1(b)) [120, 121].

An ANR-like signalling centre is also present in hemichordate embryos (Figure 1(d)). The Wnt inhibitor *sfrp1/5* as well as two* fgfs* are expressed anteriorly in *Saccoglossus*. Inhibition of *Fgf* signalling causes an anterior-to-posterior transformation whereas inhibition of Wnt signalling results in anteriorisation of marker gene expression in the proboscis [10]. Interestingly, *sfrp1* is also expressed in the animal region of the embryo of the sea urchin *Strongylocentrotus purpuratus* [122], where it may serve to protect the neurogenic animal pole domain against posteriorising Wnt signalling [123].

As yet, there is no evidence to support the existence of an ANR-like signalling centre in protostomes. However, similarities in the underlying expression of patterning genes and neuron types has led to the proposal that the anteriorly located mushroom bodies of annelids are homologous to the dorsal telencephalon of vertebrates (pallium) and at least some of the underlying patterning mechanisms seem to be conserved [124].

## 4. Dorsoventral Patterning

### 4.1. Notochord and Prechordal Plate

The notochord, a rod of dorsal mesoderm derived from Spemann's organiser/the node, is one of the defining features of chordates and a well-known organiser of DV neural patterning [125–128]. In vertebrates, a somewhat broader mesendodermal tissue that has slightly different signalling properties—the prechordal plate—is found at the anterior end of the notochord [117]. The notochord and prechordal plate are crucial for the induction of ventral CNS identity and Shh is the main signalling factor mediating this process, although Tgf*β*s and Bmp antagonists may also contribute. The induction of motor neurons throughout the ventral neural tube is one of the hallmarks of Shh function (Figure 1(a)) [127–129].

*Ciona* and amphioxus both display a notochord, but no evidence for a prechordal plate is found in either of those species [130–132]. In amphioxus, the notochord extends to the anterior tip of the embryo and amphioxus *goosecoid*, a marker of the prechordal plate in vertebrates, is only transiently expressed in cells that involute during gastrulation [133]. *Hh* and *hnf3*, markers of the notochord of vertebrates, are expressed in the notochord of amphioxus [108, 134], but surprisingly not in that of *Ciona* (Figures 1(b) and 1(c)) [109]. A recent study found that the induction of motor neurons in *Ciona* is driven by Bmps—factors that antagonise motor neuron formation in vertebrates—rather than Hh [135]. In addition to differences in gene expression, functional differences have been noted between the notochord of vertebrates and that of some of their sister groups: for example, muscle fibres are found in the amphioxus notochord as it serves a motile function [136].

Hemichordates do not form a notochord, but structural similarities exist between the chordate notochord and the hemichordate stomochord. However, orthologues of the vertebrate notochord markers *Brachyury* and *Hnf3* are not detected in this structure at any time [137–139]. There is also little evidence for a notochord-like structure in protostomes. The ventral midline of the spider embryo expresses *short gastrulation* (*sog*, the orthologue of the vertebrate Bmp antagonist *Chordin*), and this linear source of Bmp inhibition is essential for the specification of ventral structures [140]. However, this band of cells is ectodermal rather than the mesodermal notochord of chordates. *Drosophila sog* is expressed in a much broader domain throughout the ventral half of the early embryo and its requirement in ventral specification is more limited (Figure 2) [46, 141].

Taken together, there is little evidence for the presence of a structure homologous to the notochord outside of the chordate lineage. Even within the chordates, a signalling function has only been demonstrated for vertebrates, and the lack of *hh* expression in *Ciona* suggests that there is considerable flexibility as to its organiser properties. A prechordal plate is only found in vertebrates (including the jawless agnathans [107]), so one could speculate that the specific neural patterning properties of this tissue have evolved with the rapidly increasing complexity of the forebrain in this clade.

### 4.2. Floor Plate and Ventral Patterning Centres in the Forebrain

In vertebrates, the floor plate, a specialised stripe of non-neural cells running along the ventral midline of the neural plate/tube, also functions as an organiser that regulates ventral neural patterning and axon guidance by expressing *Shh* and other factors such as the axon guidance factor Netrin [125–128]. The exact mechanism of floor plate formation remains somewhat controversial, with some researchers advocating a model whereby both notochord and floor plate are derived from a common progenitor pool in Spemann's organiser/the node, whereas others favour a model according to which the notochord induces the floor plate. Furthermore, the initial view that notochord-derived Shh is the primary signal in floor plate induction had to be revised when Tgf*β*s of the Nodal family were found to be essential for this process in zebrafish embryos [125, 142–144].

Although absent from the notochord, *Ciona hh* is expressed ventrally throughout the nerve cord (Figures 1(b) and 2) [109]. Surprisingly, these floor plate-like cells are not involved in motor neuron induction [135]. In amphioxus, the floor plate expresses *hh*,* hnf3*, and *netrin*, but it remains to be established whether the cephalochordate floor plate exerts any signalling and/or axon guidance functions (Figures 1(c) and 2) [108, 134, 145]. Various components of the Hh signalling pathway are expressed in the nerve cord of amphioxus and in particular the Hh target gene *patched* becomes downregulated dorsally, possibly reflecting an involvement of this signalling pathway in neural ventralisation comparable to vertebrates [146]. Unlike vertebrates that transduce Shh signals via the combinatorial activity of three *Gli *genes, amphioxus only has a single *gli* gene that produces two transcripts with opposing activities and is expressed in the floor plate, but not in the notochord, consistent with a unidirectional induction of the lancelet's floor plate by notochord-derived Hh [147].

There is no evidence for a floor plate-like signalling centre in hemichordates. In fact, the expression of *hh* differs dramatically even between different hemichordate lineages. Interestingly, an amino acid exchange in the normally well-conserved proteolytic cleavage site of Hh reduces the signalling capacity of this factor in *Saccoglossus*, suggesting a partial degeneration of this signalling pathway in the hemichordate phylum [148].

In all protostomes analysed thus far, *hh* is expressed in a segmental fashion rather than along the midline [149–151]. However, the mechanisms that guide axons across the midline are surprisingly conserved between *Drosophila* and vertebrates [152–154]. Thus, although a *hh*-expressing floor plate seems to be specific for chordates—and may have degenerated in tunicates—the regulation of axon guidance by a midline signalling centre could be a widespread bilaterian feature.

Ventral patterning in the vertebrate forebrain somewhat differs from that in the rest of the neural tube: the prechordal plate is known to be essential for ventral forebrain induction, but Shh alone is insufficient to mediate this function [155, 156]. Bmp7 is transiently expressed in the prechordal mesoderm and may cooperate with Shh in forebrain ventralisation [155]. Furthermore, the prechordal plate expresses Wnt inhibitors that are also likely to contribute to this process [117, 157].

Once ventral identity has been established, Shh is expressed in the diencephalic basal plate, throughout most of the hypothalamus and in a ventroanterior area known as the lamina terminalis that is located just ventral to the ANR and gives rise to the medial ganglionic eminence (MGE, Figure 1(a)) [158]. This Shh expression domain, which is thought to function in the ventral specification of the telencephalon, is absent in lampreys [106, 107]. The lack of this secondary organiser in agnathans, together with the absence of several other MGE characteristics such as the expression of *nkx2.1* and the migrating interneurons derived from this area, suggests that the MGE is an evolutionary innovation in gnathostomes, possibly reflecting a requirement for increased motor coordination in higher vertebrates. Surprisingly, *hh* is expressed in a small patch in the ANR-like area of *Saccoglossus* (Figure 1(d)) and inhibiting its function using RNA interference resulted in a downregulation of the expression of one of the *Fgfs* that are normally expressed there, suggesting crossregulation of anterior patterning signals as in vertebrates [10].

### 4.3. Roof Plate

Similar to the ventral groups of neurons in the developing vertebrate neural tube, dorsal neurons are patterned by signals from a secondary organiser, the roof plate, which is established where the two lateral edges of the neural plate fuse dorsally to form the neural tube (Figures 1(a) and 2). The signalling mechanisms from the roof plate are less well defined than that of other organisers such as the floor plate; several Bmps and Wnts are expressed there, but experiments disrupting any of these signalling factors individually have led to varying results, probably due to functional redundancy [159, 160].

The character of the roof plate changes along the AP axis of the vertebrate neural tube. For example, Bmps are required in the anterior hindbrain to initiate development of the dorsal-most population of cells in this region, cerebellar granule cells, and the cerebellum of mice lacking a functional roof plate is much smaller [161, 162]. At the rostral end of the mouse neural tube, the roof plate invaginates to form the choroid plexus and cortical hem, the latter of which functions as a secondary organiser required for the induction and patterning of the hippocampus [163]. Here, the role of Bmp signalling appears to be limited to the induction of the nonneural choroid plexus [164].

There is little evidence for a Bmp- and Wnt-secreting roof plate in nonvertebrate embryos (Figure 2). In *Ciona* and amphioxus, Bmp signalling is involved in the specification of peripheral sensory neurons, similar to vertebrates where these neurons are derived from the placodes and neural crest at the border of the neural plate [165–169]. In the tunicate *Halocynthia roretzi*, *bmp2/4* remains expressed in some cells along the border of the neural plate throughout gastrulation and neurulation—these cells could be the tunicate equivalent of a roof plate, but a function in neural patterning remains to be established for them [170].

*Saccoglossus *lacks the centralised nerve cord of chordates; however, expression of *bmp2/4/5/8* and *chordin* is observed in longitudinal stripes on opposite sides of the embryo, in line with a conserved Bmp gradient patterning the DV axis of bilaterian embryos (Figures 1(d) and 2). Several genes—a few of those orthologous to vertebrate neural patterning genes—are under the control of this Bmp gradient [171]. In the *Drosophila* nerve cord, the homeobox genes *ventral nervous system defective *(*vnd*),* intermediate neuroblasts defective *(*ind*) and* muscle segment homeobox *(*msh*), mark ventral, intermediate and dorsal columns and this pattern is also regulated by the Bmp gradient that patterns the DV axis of the embryo [172]. Similarly, longitudinal progenitor domains are under the control of Bmp signalling in the annelid *Platynereis* [173]. These studies support the view that the ancient Bmp gradient mechanism that regulates DV organismal patterning is present throughout the animal kingdom, and it became involved in the patterning of the DV neuraxis secondarily. However, the presence of a secondary Bmp-secreting organiser along the dorsal midline of the CNS appears to be a chordate novelty.

## 5. Secondary Organisers in Closely Related Species

So far, we have focused on differences between animal lineages that diverged hundreds of millions of years ago. Yet, brain morphology can differ substantially, even between closely related vertebrate species. Are these morphological differences related to changes in secondary organiser function? The Mexican tetra *Astyanax mexicanus* exists in two dimorphic forms: a sighted surface fish and a blind cavefish. Although both retina and lens are induced in embryos of *Astyanax*, they degenerate subsequently and undergo apoptosis. These alterations are due to changes of ANR signalling, where *Fgf8* expression is initiated approximately two hours earlier in the cavefish embryos compared to their surface counterparts, due to an expanded domain of Shh expression in the ventral forebrain [174, 175]. Strikingly, eye development can be restored by treating cavefish embryos with a pharmacological Fgf inhibitor. These studies highlight that a small heterochrony in secondary organiser signalling can have dramatic effects even within one species.

Cichlid fishes are renowned for their rapid evolution and diversity, and the wide variation seen in their brain morphology is correlated to their various habitats [176]. Recent work has demonstrated that this morphological complexity can be traced back to differences in secondary organiser activity early in embryonic development. The relative size of forebrain subdivisions differs between rock-dwelling, and sand-dwelling cichlids from Lake Malawi. For example, the rock-dwelling species display a more elaborate diencephalon and larger thalamus. These size changes correspond to subtle differences in secondary organiser signalling: the expression of Wnt1 at the MHB is stronger in the rock-dwellers and the angle of the ZLI in both species is different. Notably, these alterations can be mimicked by subtle experimental manipulation of Wnt signalling levels [177]. These two studies highlight that changes in secondary organiser activity can drive morphological diversity in closely related, or even in the same species.

## 6. Concluding Remarks

The molecular patterning systems that regionalise animal embryos along their primary and secondary axes are highly conserved and may have existed before the development of bilateral symmetry. Secondary organisers that fine-tune the development of the nervous system seem to be a more recent acquisition. Different combinations of secondary organisers are present in different phyla (Figures 1 and 2), but the recent finding that signalling centres strongly resembling the MHB, ZLI and ANR are present in a hemichordate embryo suggests that they have evolved earlier than previously thought, and that they may have been present in the deuterostome ancestor. These findings also suggest that secondary organisers are not inherently linked with neuroepithelial identity, since the acorn worm analysed in the hemichordate study does not possess a central nerve cord like chordates but a diffuse nerve net. However, some degree of nervous system centralisation has also been described in hemichordates [99]. Therefore, it will be crucial to investigate more species within this phylum—as exemplified by a recent study in *Balanoglossus simodensis* [100]—to obtain a more complete picture as to how the development of their nervous system relates to other clades.

Why are some of the signalling centres found in hemichordates absent from true chordates such as the cephalochordate amphioxus or the tunicate *Ciona* that are more closely related to vertebrates? One possible answer to this question is that these lineages have lost some of the secondary organisers in the course of evolution, as an adaptive mechanism that drove changes in CNS morphology. Although this seems to be the most likely explanation, we cannot formally rule out that some secondary organisers have evolved independently in different lineages, for example by local derepression of a genetic module that is characteristic of a specific organiser region.

The signalling centres that regulate DV patterning in vertebrates—notochord, prechordal plate, floor plate, MGE, and roof plate—seem to have evolved more recently as they are only found in the chordate lineage. Since the DV axis is typically shorter than the AP axis, the Bmp gradient that specifies the secondary body axis early in development may be sufficient to also pattern the nervous system along this axis. Indeed, DV neural patterning is under the control of Bmp, but not Hh signalling in protostomes (Figure 2). Thus, the gradient of Bmp signalling is a good example for an ancient patterning system that has been coopted secondarily to regulate DV patterning of the nervous system.

So far, evidence for secondary organisers in protostomes is very limited. This poses the question whether a complex nervous system can form without signalling centres. Octopoda, an order within the Lophotrochozoa, represent a special case amongst invertebrates due to their highly complex nervous system and high cognitive abilities. We expect that an investigation of octopus development will provide some fascinating insights into the role of local signalling centres in neural patterning. It is important to keep in mind that all research into secondary organisers so far has been heavily biased towards vertebrates—it is very well possible that nonvertebrates feature sets of completely unrelated secondary organisers.

In conclusion, our understanding of the phylogenetic origins of secondary organisers is still a puzzle with many missing pieces. In the future, it will be important to compare the expression of signalling and patterning genes in many more species within each phylum. The species that have been used for experimental approaches have often been chosen for their specific advantages in the lab such as a short generation time; thus, they may not always be most representative for a specific phylum.

The absence of some secondary organisers in closer relatives of the vertebrates, combined with the observation that subtle changes in organiser function underlie morphological differences between closely related species suggest a relative evolutionary flexibility of these signalling centres. Recently, another type of (nonevolutionary) flexibility was demonstrated in the mouse embryo: the otherwise Shh-expressing ZLI turns into an Fgf8-expressing ring in *Shh*;* Gli3* double-mutant mice, suggesting that signal factor expression can be uncoupled from the positional identity of a secondary organiser to some extent [178]. 

A common feature of secondary organisers in vertebrates is that they form along boundaries that are stabilised by Notch signalling and that are characterised by reduced proliferation and high levels of *Hes/Hairy* gene expression [9, 179]. So far, these features have not been studied in much detail at local signalling centres of nonvertebrates.

Based on the comparative studies that we have reviewed here, it appears that a full complement of secondary organisers is not required to generate a functional nervous system. So, what is their phylogenetic relevance? Francois Jacob published an article on “Evolution and tinkering” in Science in 1977, where he proposed that evolution is likely to progress more rapidly by the opportunistic rearrangement of preexisting elements (“*bricolage*”), rather than the constant de novo creation of new elements [180]. We propose that, at least in the deuterostome lineage, secondary organisers are mediators, *bricoleurs*, of such a process whereby small changes in signalling strength, signal identity, relative position, or even the presence or absence of a signalling centre generates morphological complexity from the comparably stable tripartite ground plan.
